# The Possible Detriment of Oxygen in Creep of Alumina and Zirconia Ceramic Composites Reinforced with Graphene

**DOI:** 10.3390/ma14040984

**Published:** 2021-02-19

**Authors:** Rafael Cano-Crespo, Pedro Rivero-Antúnez, Diego Gómez-García, Rodrigo Moreno, Arturo Domínguez-Rodríguez

**Affiliations:** 1 Departamento de Física de la Materia Condensada, Universidad de Sevilla, Apartado 1065, 41080 Sevilla, Spain; privero@us.es (P.R.-A.); dgomez@us.es (D.G.-G.); adorod@us.es (A.D.-R.); 2 Instituto de Ciencia de Materiales de Sevilla, CSIC-USE, Avenida Américo Vespucio 49, 41092 Sevilla, Spain; 3 Instituto de Cerámica y Vidrio, CSIC, 28049 Madrid, Spain; rmoreno@icv.csic.es

**Keywords:** ceramic, composite, high temperature, microstructural characterization, plasticity

## Abstract

This paper aims to give an answer to the following question: is the oxidation of graphene a critical issue for high-temperature plasticity in graphene-reinforced ceramics? To give a convincing reply, we will focus on two very different graphene-based ceramic composites: reduced graphene oxide (rGO)-reinforced alumina (α-Al_2_O_3_) and reduced graphene oxide (rGO)-reinforced yttria tetragonal zirconia (t-ZrO_2_). The processing of the powders has been made using a colloidal route, and after that, a spark plasma sintering process was performed in order to densify the samples. Creep tests were performed at temperatures between 1200–1250 °C in an argon atmosphere. The microstructure obtained by SEM of the sintered and tested specimens was characterized quantitatively to elucidate the deformation mechanism. Raman spectroscopy was carried out to check the integrity of the graphene. The average grain size was in the order of 1 µm and the shape factor was 0.7 for all the studied materials. The integrity of the graphene was checked before and after the creep experiments. The careful analysis of the creep tests shows that graphene oxide or its reduced version are not efficient phases for creep resistance improvement in general, contrary to what is reported elsewhere. However, the results permit the suggestion of a creep improvement in nanocomposites at a very high temperature regime due to an enhanced reactivity of oxygen between carbon and alumina interfaces. In the case of zirconia, the results give us the conclusion that the oxidation of graphene is a highly detrimental issue regarding the improvement of high-temperature plasticity.

## 1. Introduction

Traditionally, a secondary phase is used in the world of materials to reinforce them and to try and change its mechanical properties. The result is a composite with enhanced properties [1,2]. In the last few years, carbon materials such as carbon nanotubes (CNTs) [3], carbon fibers (CFs) [4], carbon nanofibers (CNFs) [5], graphite, graphene (G) [6,7] or graphene oxide (GO) [6] have become very interesting materials due to their very good properties and the employment of them to optimize the mechanical properties. For example, in the case of graphene, Yang et al. [8] fabricated carbon/graphene/carbon composites and found an improvement of the mechanical properties (flexural strength, interlaminar shear strength, interfacial debonding strength, internal friction and storage modulus) compared to the carbon/carbon composite. These authors also found that these properties were strongly influenced by the fiber/matrix interface which was modified by graphene. One important use of these new materials is as a reinforcement phase of advanced ceramic materials at high temperatures [9].

Concerning alumina, (Al_2_O_3_) there are a lot of studies trying to improve the creep behavior by adding a secondary phase. For example, Lessing et al. [10] found that the pure material has worse mechanical properties than alumina doped with transition metal ions. Weidner et al. [11] studied the high temperature mechanical properties of alumina-based composites reinforced with a 11 or 21 vol.% of refractory metal Ta and Nb, respectively. These materials did not fail in a completely brittle manner during compression tests between 1300–1500 °C. Tamura et al. [12] sintered by spark plasma sintering an alumina-whisker-reinforced alumina composite and achieved an improvement of the creep resistance by around one order of magnitude compared to the pure material. On the other hand, Zapata-Solvas et al. [13] and Padture [14] found that Al_2_O_3_ doped with a 10 vol.% of carbon nanotubes had a better creep behavior than the pure material with a similar microstructure. In another study, Zapata-Solvas et al. [15] found an improvement of the creep resistance in Al_2_O_3_/single-wall carbon nanotube composites. An extensive variety of alumina exists, with a large scatter in the diffusion kinetics processes which can also affect the mechanical properties [16,17,18]. More recently, Cano-Crespo et al. [19] sintered by spark plasma sintering carbon nanofibers reinforced with alumina and graphene oxide reinforced with alumina composites, respectively. Using a concentration of 2 vol.%, they studied the creep at high temperatures and at 1200 °C the creep resistance of the Al_2_O_3_/ carbon nanofiber composite decreased with respect to pure alumina, but at 1250 °C they found a similar behavior of the composite and the pure material. The same authors reported that the Al_2_O_3_/graphene oxide composite has a better creep resistance than the pure material. Zapata-Solvas et al. [20] employed graphite as a secondary phase using a concentration of 10 vol.% and found an improvement of creep resistance.

In the case of zirconia, several researches have been performed in the field of the study of the creep behavior of zirconia-reinforced composites. Grain boundary sliding (GBS) is the most important mechanism of deformation at high temperatures of yttria-stabilized-tetragonal zirconia (3YTZP) [21,22]. Some authors [23,24] reported research about the creep behavior of zirconia reinforced with ceria. Calderón-Moreno et al. [25] sintered a composite alumina/zirconia and studied its creep behavior. Lorenzo-Martín et al. [26] found that grain boundary sliding was the predominant deformation mechanism in a composite of zirconia with a glassy phase, and they also found that the creep rate depended critically on the content of the secondary phase added. Whiskers of SiC have been employed for years, as was the case of Calderón-Moreno et al. [27]; these authors sintered and researched the mechanical properties of ZrO_2_-Al_2_O_3_/SiC composites at high temperatures. A review summarizes the most important features of the research in carbon nanotube reinforced zirconia composites [28]. Cano-Crespo et al. [29] found that carbon nanofiber reinforced zirconia composites were less creep resistant than the same material without the secondary phase. The same authors also reported that graphene oxide reinforced zirconia composites were systematically less creep resistant than pure zirconia. The difference between this study and the present study is the secondary phase employed. In the previous study, graphene oxide was used in order to reinforce zirconia, but in the present study, reduced graphene oxide, a graphene with some defects in its lattice, has been used in two different concentrations to check the possible change in the creep behavior.

The scientific problem is the comprehension of the importance of the reactivity of oxygen between the graphene and the alumina or zirconia grains, and if this reaction is detrimental in the mechanical properties at high temperatures. In order to give an answer, several creep experiments at very elevated temperatures using graphene as the secondary phase were performed for the first time of our knowledge. Consequently, the main goal of this study is to prepare a homogeneous dispersion of reduced graphene oxide (rGO) into alumina and zirconia matrices and to obtain nearly full density compacts by spark plasma sintering with enhanced creep resistance at high temperatures compared to pure materials. To achieve this aim, several experimental techniques were employed: scanning electron microscope, Raman spectroscopy, and creep at high temperatures. The creep experiments were analyzed according to the Equations (1)–(3) presented in the Section 2.5.

## 2. Experimental Procedure

### 2.1. Starting Materials

The starting powders of α-alumina and tetragonal zirconia used were the same as in [19,29]. The powder of α-Al_2_O_3_ (Celarox, Condea HPA05, Sasol, Lake Charles, LA, USA) has an average particle size of 0.35 µm and a specific surface area of 9.5 m^2^/g. The powder of tetragonal-ZrO_2_ stabilized with 3% mol of Y_2_O_3_ (TZ-3YSE, Tosoh, Tokyo, Japan) has an average particle size of 90 nm and a specific surface area of 6.7 m^2^/g. Both kinds of powders have a high purity and higher than 99.9%.

Reduced graphene oxide (Nanoinnova Technologies, Toledo, Spain) with a length of 1–4 μm, a thickness of 0.7–1.2 nm and a surface area of 103 m^2^/g was used as the secondary phase [30,31]. Particle sizes were measured by laser diffraction using a Mastersizer S apparatus (Malvern, UK) and the surface area was measured by single point adsorption (Monosorb, Quantachrome, Boynton Beach, FL, USA).

### 2.2. Powder Processing Methods

First of all, reduced graphene oxide was dispersed in **N**-methyl-2-pyrrolidone (NMP) with magnetic stirring, as this is considered elsewhere an excellent non-aqueous vehicle to achieve the highly efficient dispersion of reduced graphene oxide. Alumina was dispersed in water using an ammonium salt of poly(acrylic) acid (Duramax D3005, Rohm and Haas, Dow Chemicals, Midland, MI, USA) in a concentration of 0.5 wt.% on a dry solids basis and sonicated with different sonication times. Concentrated suspensions of alumina-reduced graphene oxide were prepared to solid loadings of 30 vol.% and homogenized with 1 min of ultrasonication.

To obtain a homogeneous dispersion of reduced graphene oxide in zirconia, both materials were dispersed independently and furtherly mixed. Zirconia was dispersed in water to a concentration of 30 vol.% using an ammonium salt of poly(acrylic) acid (Duramax D3005, Rohm and Haas, Dow Chemicals, Midland, MI, USA) in a concentration of 0.5 wt.% on a dry solid basis. Reduced graphene oxide was dispersed in 1-methyl-2-pyrrolidone at the same solids content (30 vol.% reduced graphene oxide) and both slurries were subjected to mechanical homogenization and sonication for 1 min (Dr. Hielscher UP400S, Teltow, Germany). Once dispersed, proper fractions of both suspensions were mixed altogether to obtain two relative contents of reduced graphene oxide in the zirconia matrix of 2 and 6.7 vol.%, applying 1 min of additional sonication. The rheological behavior of the different suspensions was analyzed using a rotational rheometer.

The optimized suspensions of the mixture were frozen in a rotatory chamber using liquid nitrogen as refrigerant (−196 °C). The frozen suspensions were introduced in a freeze-dryer for 24 h. The condensator temperature was −50 °C, and the conditions of the storage camera were 20 °C and 0.050 mbar [30].

The following resultant mixtures of powders were obtained:

- A2rGO: 98 vol.% α-alumina and 2 vol.% reduced graphene oxide.
- A6.7rGO: 93.3 vol.% α-alumina and 6.7 vol.% reduced graphene oxide.
- Z2rGO: 98 vol.% t-zirconia and 2 vol.% reduced graphene oxide.
- Z6.7rGO: 93.3 vol.% t-zirconia and 6.7 vol.% reduced graphene oxide.

As reference materials, pure alumina and pure zirconia commercial powders were used for the sake of comparison. These powders are named A and Z, respectively.

### 2.3. Sintering

The sintering process employed was the same as in [19,29]. The consolidation of the samples was performed in a spark plasma sintering machine and employed a vacuum in order to prevent the damage of the carbon phase. The resultant mixtures of powders were introduced in a graphite die. The maximum temperature was 1300 °C, using a holding time of 5 min. The applied pressure during all the process was 75 MPa and the heating and cooling rate were 100 °C/min, respectively.

### 2.4. Microstructural Characterization

Raman spectroscopy was performed in order to note the integrity of the reduced graphene oxide. Measurements were performed in the same way as described in [19,29].

The microstructural characterization was performed by scanning electron microscopy. The surfaces of the samples were polished with diamond paste. The microstructure was described by measuring the grain size and the shape factor. Fracture surfaces were also observed to check the presence and distribution of reduced graphene oxide; to this end a high resolution scanning electron microscope (Model FEI TENEO, Hilsboro, Washington, OR, USA) was employed in the mode secondary electrons.

### 2.5. High-Temperature Mechanical Tests

The creep at high temperatures of these new materials was investigated in the same way as explained in [19,29]. It measured the stationary strain rate as a function of the stress (the applied load) and the temperature. In general, the creep experiments obey the following phenomenological equation:

(1) $$\dot{\varepsilon }=A\frac{Gb}{kT}{\left(\frac{b}{d}\right)}^{p}{\left(\frac{\sigma }{G}\right)}^{n}D$$

where $\dot{\varepsilon }$ is the strain rate, *A* is a dimensionless constant, *G* is the shear modulus, *b* is the magnitude of the Burgers vector, *k* is the Boltzmann constant, *T* is the absolute temperature, *d* is the grain size, *p* is the inverse of the exponent of the grain, *σ* is the stress, *n* is the stress exponent and *D* a diffusion coefficient [32].

The characteristics parameters of the creep experiments were the stress exponent (*n*) and the activation energy (*Q*). A value of stress exponent was obtained by changing the applied load as it can be seen in the Equation (2) and a value of the activation energy was obtained by modifying the temperature (3).

(2) $$n={\left[\partial \left(ln{\dot{\varepsilon }}_{ss}\right)/\partial \left(ln\sigma_{ss}\right)\right]}_{T}=\left(ln\frac{{\dot{\varepsilon }}_{2}}{{\dot{\varepsilon }}_{1}}\right)/\left(ln\frac{\sigma_{ss2}}{\sigma_{ss1}}\right)$$

(3) $$Q_{c}=-k{\left[\partial \left(ln{\dot{\varepsilon }}_{ss}\right)/\partial \left(1/T\right)\right]}_{\sigma_{ss}}\approx -k\left(\frac{ln\frac{{\dot{\varepsilon }}_{1}}{{\dot{\varepsilon }}_{2}}}{\frac{1}{T_{1}}-\frac{1}{T_{2}}}\right)$$

In the previous equations *ss* denotes the stationary state. The measuring of *n* and *Q*, together with the characterization of the microstructure, give us an idea of the predominant deformation mechanism in the material.

A chamber containing argon was employed in all the experiments to avoid the combustion of reduced graphene oxide. Two values of temperature were used: 1200 °C and 1250 °C. The interval of stress applied was between 9–300 MPa.

## 3. Results and Discussion

### 3.1. Characterization of the Mixtures of Powders

**Figure 1:**
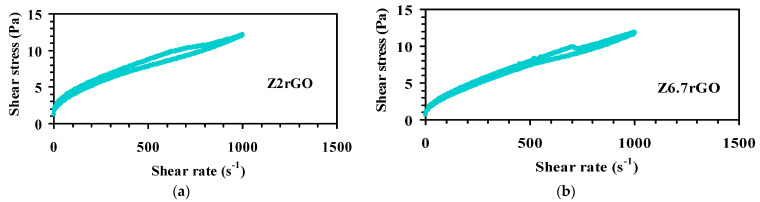
Flow curves of 30 vol.% solids suspensions of zirconia with (**a**) 2 vol.% of reduced graphene oxide (rGO) and (**b**) 6.7 vol.% of rGO. For both suspensions the shear stress (Pa) is plotted versus the shear rate (s^−1^).

In a first step, the powders of the mixtures were prepared. The starting suspensions of alumina with reduced graphene oxide showed a Newtonian behavior and very low viscosity (7 mPa·s) demonstrating their good dispersibility. Zirconia was dispersed in water, whereas reduced graphene oxide was dispersed in a non-aqueous medium (NMP) in order to improve the dispersion. The final suspensions had a solid loading of 30 vol.%. Figure 1 shows the rheological behavior for the suspensions of zirconia with reduced graphene oxide (Z2rGO and Z6.7rGO). The slope in the two cases is almost constant. The curves exhibit a slightly shear thinning behavior with a small thixotropy and viscosity values at a maximum shear rate of around 12 mPa·s, low enough to ensure stability. Those suspensions were freeze dried to obtain the powders of the reduced graphene oxide/zirconia mixtures. After the spark plasma sintering process, all the samples were fully dense with a value of density near-to theoretical density, measured by the Archimedes method with distilled water, higher than 99 % and consequently they were optimal to study their mechanical properties at high temperatures (creep experiments).

### 3.2. Microstructural Characterization

**Figure 2:**
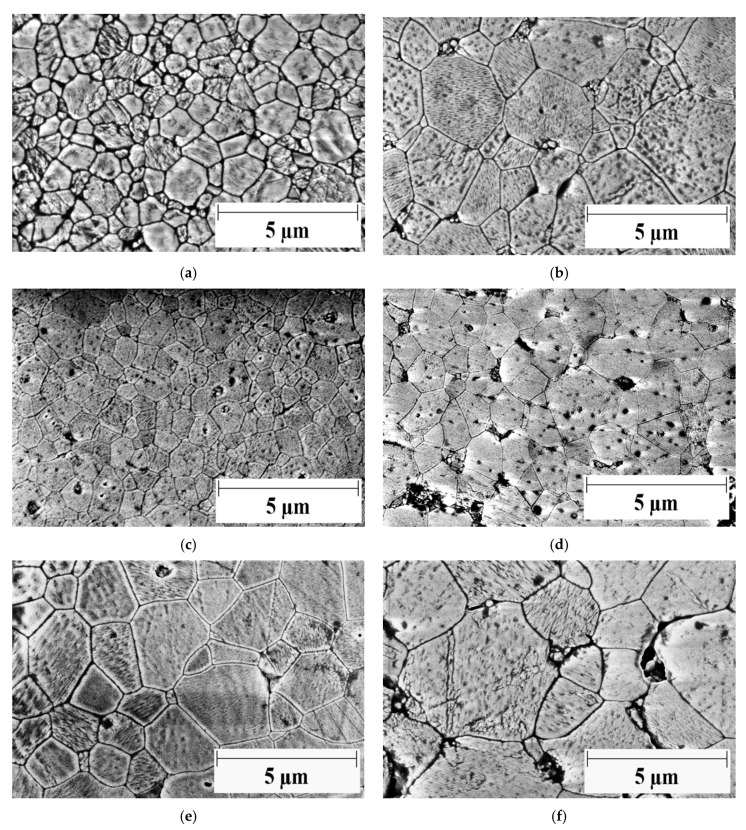
HRSEM (High resolution scanning electron microscopy) micrographs of (**a**) as-sintered monolithic Al_2_O_3_, (**b**) monolithic Al_2_O_3_ after creep, (**c**) as-sintered 98 vol.% Al_2_O_3_–2 vol.% rGO, (**d**) 98 vol.% Al_2_O_3_–2 vol.% rGO after creep, (**e**) as-sintered 93.3 vol.% Al_2_O_3_–6.7 vol.% rGO and (**f**) 93.3 vol.% Al_2_O_3_–6.7 vol.% rGO after creep. The same scale was used for all the cases, 5 µm. The accelerating voltage of electrons was equal to 5 kV.

Figure 2 displays the thermal etched surfaces of A, A2rGO and A6.7rGO before and after creep experiments at high temperatures. The grain boundaries between the grains can be seen, a typical image of a ceramic polycrystal. Some little holes can be appreciated due to the polish process which removes some particles from the surface. All the images were obtained using the mode of secondary electrons and with the same scale in order to obtain a better comparison between the samples. Table 1 summarizes some magnitudes of the microstructures. The shape factors have the same value for all the cases: 0.7, which constated that the microstructure is constant during the experiments at high temperatures. Some grain growth was observed in alumina and A6.7rGO, in the first case due to the absence of the secondary phase and in the second case the reason was the high concentration of reduced graphene oxide which provoked percolation of the grains. For A2rGO, the grain size was the same before and after creep within the experimental uncertainty. Rarely, there are some grains with an abnormal size, but they are not important due to their low quantity, which means that they are insignificant to the influence on the mechanical behavior. The presence of reduced graphene oxide inhibits the grain growth at low concentrations, but at higher concentrations is not efficient to this end.

**Figure 3:**
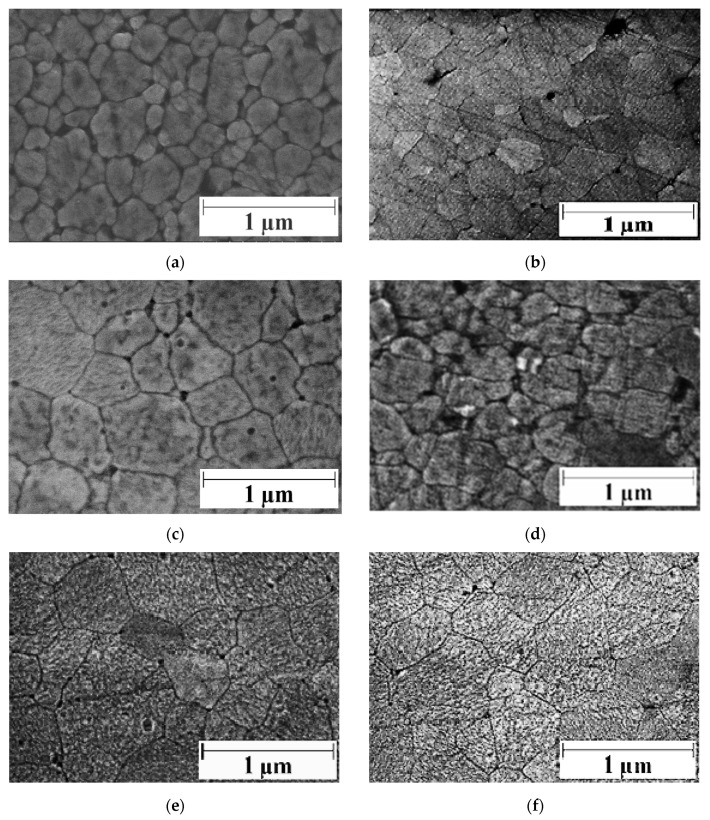
High resolution scanning electron microscopy micrographs of (**a**) as-sintered pure zirconia, (**b**) pure zirconia after creep, (**c**) as-sintered Z2rGO, (**d**) Z2rGO after creep, (**e**) as-sintered Z6.7rGO and (**f**) Z6.7rGO after creep. The same scale was used for all the cases, 1 µm. The accelerating voltage of electrons was equal to 5 kV.

**Figure 4:**
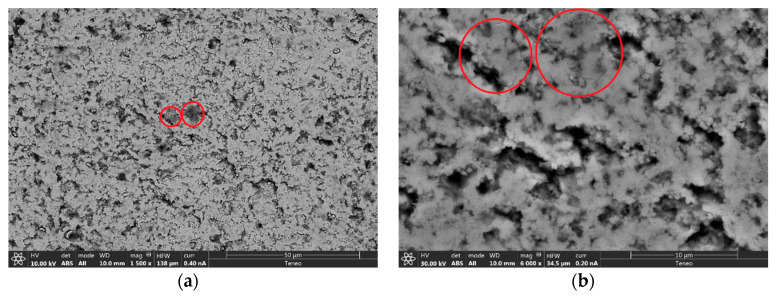
High resolution scanning electron microscopy micrographs of fracture surfaces corresponding to as-sintered Z6.7rGO. The accelerating voltage is 10 kV for (**a**) and 30 kV for (**b**). The electrical current is 0.40 nA for (a) and 0.20 nA for (**b**). The scale for (**a**) is 50 µm and for (**b**) is 10 µm. For both cases, an angular backscattered detector is employed; in other words, the detector receives the backscattered electrons. In these cases, the image is built with all the surface of the detector, removing the topographic contrast and enhancing the contrast due to the difference in the atomic number.

Figure 3 displays the thermal etched and polished surfaces of zirconia, Z2rGO and Z6.7rGO, before and after creep experiments. The same facts were observed as in Figure 2: the grain boundaries and the little holes. Again, the mode of secondary electrons and the same scale were employed for a better comparison. The average grain sizes have values between 0.2–0.3 μm and the shape factor is also the same and equal to 0.7, which means that the microstructure was independent of the secondary phase added and constant during creep experiments. Figure 4 shows high resolution scanning electron microscopy images of fracture surfaces corresponding to Z6.7rGO as sintered; it can be seen surrounded by red circles with some laminates of reduced graphene oxide. From these images, it can be deduced that the distribution of reduced graphene oxide around all the microstructure is optimum and another important fact is the absence of agglomerates.

**Figure 5:**
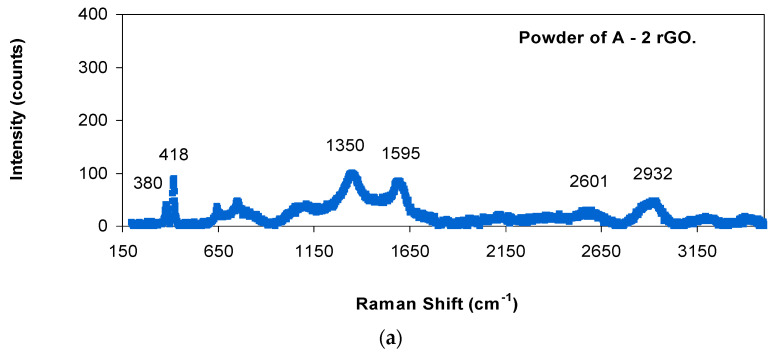

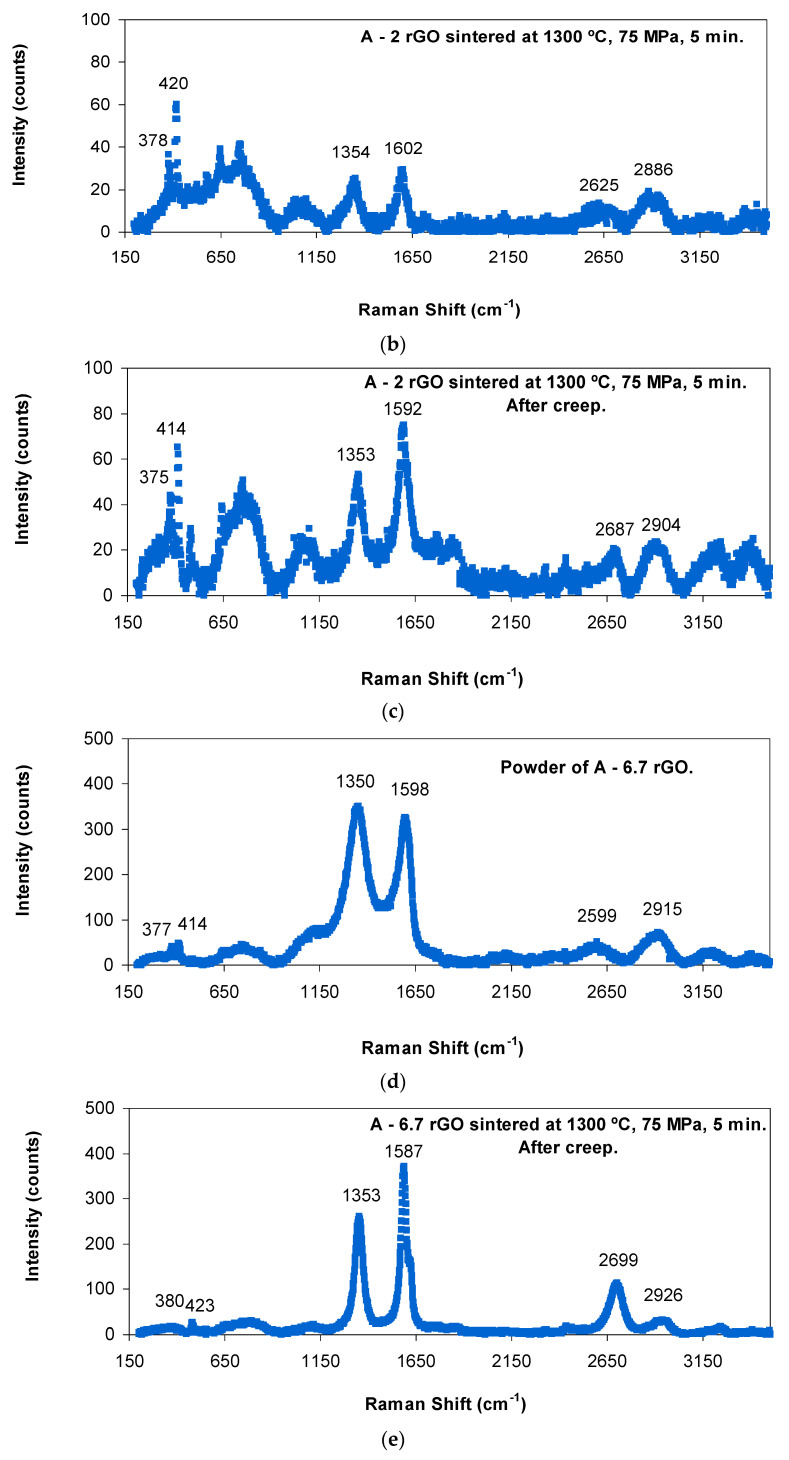

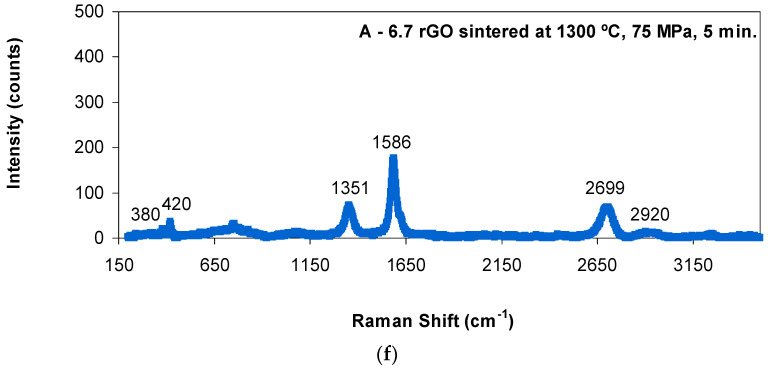
Raman spectra of (**a**) powder of 98 vol.% Al_2_O_3_–2 vol.% rGO, (**b**) sample of 98 vol.% Al_2_O_3_–2 vol.% rGO before creep, (**c**) sample of 98 vol.% Al_2_O_3_–2 vol.% rGO after creep deformation, (**d**) powder of 93.3 vol.% Al_2_O_3_–6.7 vol.% rGO, (**e**) sample of 93.3 vol.% Al_2_O_3_–6.7 vol.% rGO before creep and (**f**) sample of 93.3 vol.% Al_2_O_3_–6.7 vol.% rGO after creep deformation. It is plotted the intensity (counts) versus the Raman shift (cm^−1^).

**Figure 6:**
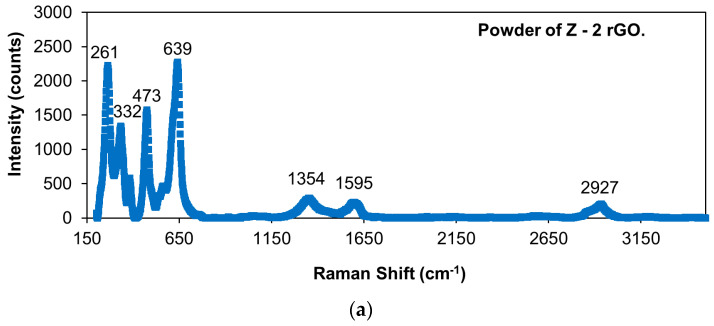

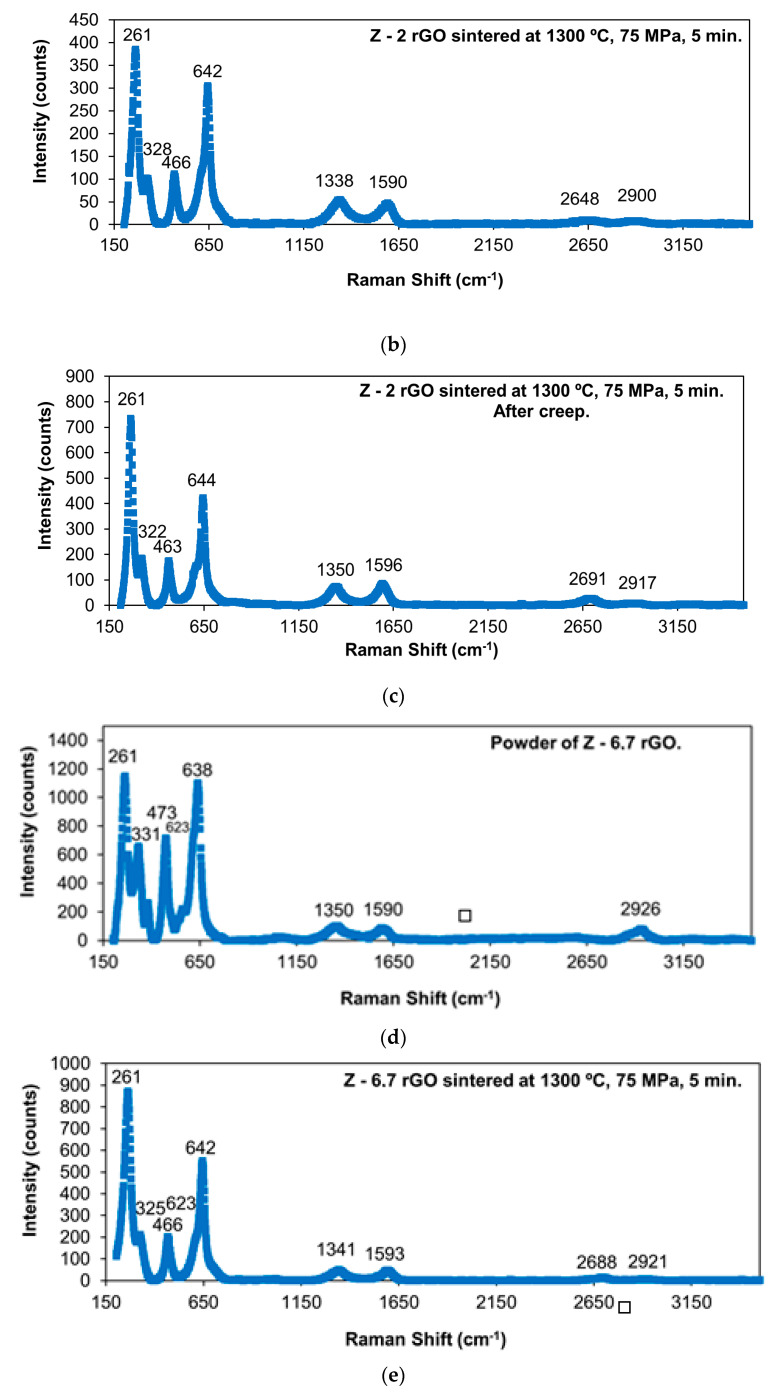

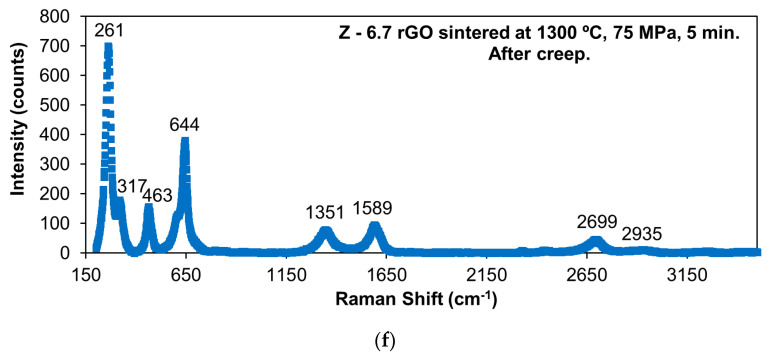
Raman spectra of (**a**) powder of Z2rGO, (**b**) sample of Z2rGO before creep, (**c**) sample of Z2rGO after creep deformation, (**d**) powder of Z6.7rGO, (**e**) sample of Z6.7rGO before creep and (**f**) sample of Z6.7rGO after creep deformation. It is plotted the intensity (counts) versus the Raman shift (cm^−1^).

Figure 5a–f shows the Raman spectra of the reduced graphene oxide-reinforced alumina materials: the powders, as sintered and after creep. Table 2 displays the positions of the peaks. The presence of the two first peaks is due to main phase of α-alumina, the next peaks correspond to the secondary phase added of reduced graphene oxide [33,34,35] and they are due to the D mode (longitudinal optical phonons), the G mode (tangential shear mode of carbon atoms) and the second order band 2D (number of layers of graphene or reduced graphene oxide), respectively. Figure 6a–f shows the Raman spectra of the reduced graphene oxide-reinforced zirconia materials: the powders, as sintered and after creep. The positions of the different peaks of the previous Raman spectra are in Table 3. The four first peaks are due to tetragonal zirconia [36,37]. The next peaks correspond to reduced graphene oxide, as explained previously. The growth in the peak at ~2700 cm^−1^ observed in Figure 5b,c,e,f and in Figure 6b,c,e,f is related to the oxidation of the carbon phase; this phase is almost intact but has oxidized at least partially [38]. It can be constated that the position of the peaks is constant. The intensity ratios between the peak D and G for the starting materials and the fabricated composites are shown in Table 2 and Table 3. This magnitude gives us an idea of the damage of the carbon phase after sintering and after creep at high temperatures. The values of the positions of the peaks from Figure 5 and Figure 6 and also from the Table 2 and Table 3 permit us to affirm that graphene remains intact during the sintering process and also during creep experiments at high temperatures, and no degradation could be observed.

### 3.3. Analysis of the Creep Experiments

**Figure 7:**
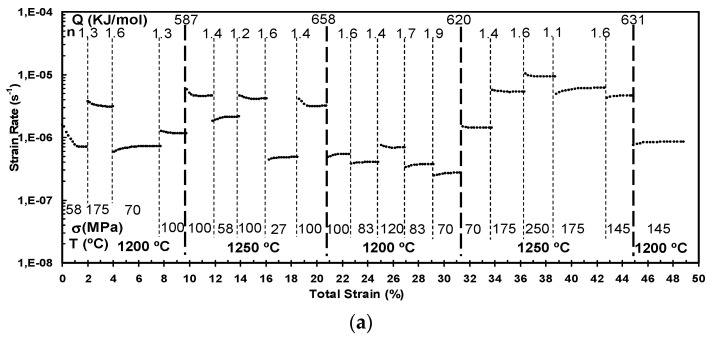

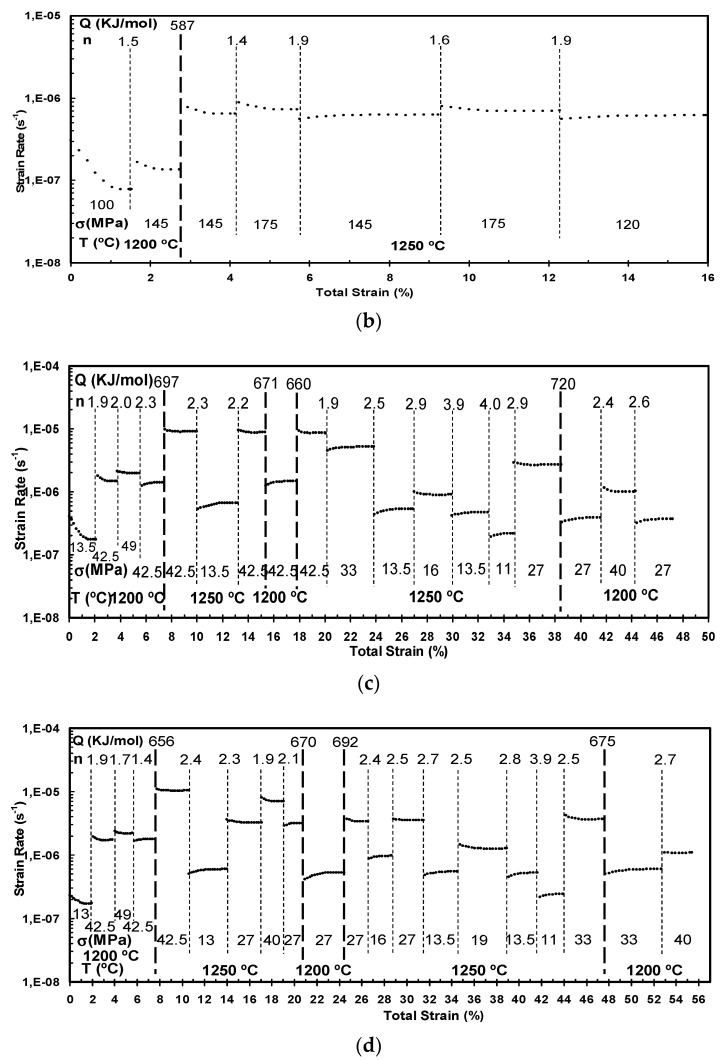
Creep curves of (**a**) 98 vol.% Al_2_O_3_–2 vol.% rGO, (**b**) 93.3 vol.% Al_2_O_3_–6.7 vol.% rGO, (**c**) 98 vol.% ZrO_2_–2 vol.% rGO and (**d**) 93.3 vol.% ZrO_2_–6.7 vol.% rGO. It is plotted the strain rate (s^−1^) versus the total strain (%). Dashed vertical lines separate the different stages in the experiments. Each stage is characterized with a value of stress (*σ* (MPa)) and a value of temperature (*T* (°C)). A value of stress exponent (*n*) is obtained with a change of the applied stress and a value of activation energy (*Q*) is also obtained if the temperature is modified. 1,E-0n denotes 10^−n^.

Figure 7 displays all the creep experiments which were performed. The stress exponents are in the interval 1.5 and 2.0 for all the composites of alumina, but for zirconia and its composites, these values are in the interval 2.5 and 3.0. The activation energy for A2rGO and for A6.7rGO are around 600 kJ/mol, which are in good agreement with the average values achieved for pure alumina polycrystals [16]. For Z2rGO and Z6.7rGO, the magnitude is around 700 kJ/mol. The reduced graphene oxide is almost intact during creep experiments at high temperatures.

All these experimental facts are in good concordance with a general model of the creep deformation in Y_2_O_3_ stabilized ZrO_2_ and in Al_2_O_3_ ceramics in which there is grain boundary sliding with diffusion as an accommodation mechanism [21,39]. The final strain of A2rGO is ~50%, in contrast, when the concentration of reduced graphene oxide is higher; in the case of A6.7rGO the final strain is in between 15–20 %. The reason can be twofold: this second type of material has a higher grain size (2.4 µm for 6.7 vol.% of reduced graphene oxide versus 1.0 µm for 2 vol.% of reduced graphene oxide) and the higher concentration of reduced graphene oxide can produce cavitation and cracks during the creep. Given the fact of the limited plasticity of A6.7rGO, several independent creep tests had to be performed to increase the number of $\dot{\varepsilon }$, *T* and *σ* data. Therefore, additional values of the stress exponents and activation energies were obtained. These facts and the same microstructure before and after creep permit us to affirm that the most important deformation mechanism is grain boundary sliding accommodated by diffusion [16,39]. The secondary phase added of reduced graphene oxide changes the grain motion, but they do not modify the diffusion, which means the accommodation mechanism [19]. The stress exponents in pure zirconia samples are approximately the same as that found in the composites reinforced with reduced graphene oxide. These experimental results, the obtained values of stress exponents and the same microstructure before and after the creep experiments permit us to say that the deformation mechanism at high temperatures must be grain boundary sliding [39]. The reduced graphene oxide seems to be a lubricant which does not limit the grain boundary mobility. The reduced graphene oxide “wets” the boundaries in a very efficient way.

**Figure 8:**
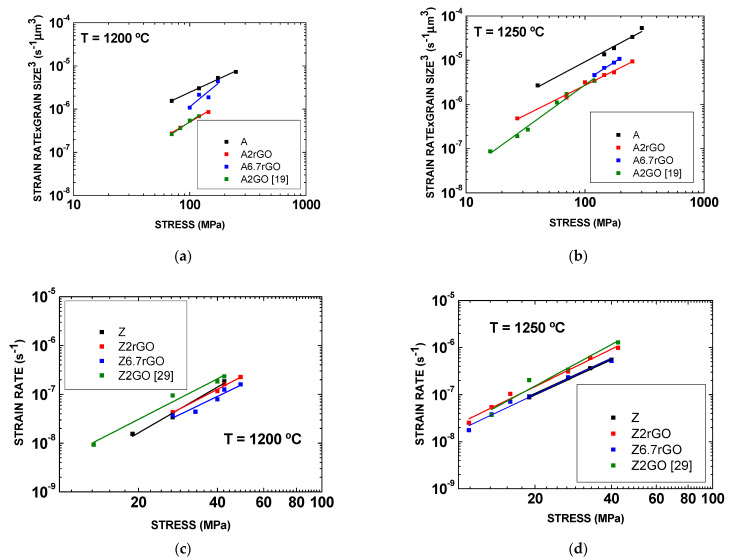
The plots of the steady-state creep rate times the cubed of the average grain size after creep versus applied uniaxial compressive stress data for A, A2rGO, A6.7rGO and A2GO [19] at (**a**) 1200 °C and (**b**) 1250 °C. The plots of the steady-state creep rate versus applied uniaxial compressive stress data for pure Z, Z2rGO, Z6.7rGO and Z2GO [29] at (**c**) 1200 °C and (**d**) 1250 °C. The stress exponents in (**a**) are: A-1.3, A2rGO-1.5, A6.7rGO-2.1 and A2GO-1.8. The stress exponents in (**b**) are: A-1.5, A2rGO-1.4, A6.7rGO-1.7 and A2GO-2.0. The stress exponents in (**c**) are: Z-3.1, Z2rGO-2.6, Z6.7rGO-2.8 and Z2GO-2.9. The stress exponents in (**d**) are: Z-2.5, Z2rGO-2.7, Z6.7rGO-2.6 and Z2GO-3.1.

One aspect which that is very important is the analysis of the creep resistance due to the introduction of this carbon phase. To this end, and assuming that the grain size exponent is three because the accommodation process is the diffusion along the grain boundary, we are using this value to normalize strain rate versus stress plots in log–log scales in Figure 8a,b for pure alumina (A), reduced graphene oxide reinforced alumina composites (A2rGO and A6.7rGO) and graphene oxide reinforced alumina composites (A2GO) from data of previous work [19]. The values for pure zirconia and its composites are in Figure 8c,d (Z, Z2rGO, Z6.7rGO and Z2GO from the literature [29]). It was not necessary to normalize the values of the strain rate due to the fact that all the samples after creep had the same grain size inside the experimental uncertainty and were equal to 0.2–0.3 μm. The experimental data fit to lines according to the equation proposed by Dorn. From these plots, it can be seen that the differences between alumina and the two composites (A2rGO and A6.7rGO) are very close, since the difference is around one order of magnitude at 1200 °C and half an order of magnitude at 1250 °C. A2rGO and A2GO have a similar creep behavior at 1200 °C and 1250 °C, being more similar than pure alumina since the difference between these composites and pure alumina is around half an order of magnitude, again inside the experimental scattering. However, there is an interesting point which must be pointed out: Figure 8a,b display that the extrapolation to the low stress range would predict a significant increase in the creep improvement. In both cases, the increment in the creep resistance must be related to the enhanced reactivity of graphene oxide with the grain boundaries of alumina, a fact which is not surprising according to the literature [40]. Should it happen, the grain mobility is restricted due to the interconnected network of carbon layers and grains. From these plots, it can be seen that all composites (Z2rGO, Z6.7rGO and Z2GO) have a similar behavior to that of pure zirconia. At 1200 °C and 1250 °C, there is the same behavior of the materials studied.

The reactivity of oxygen, present in the lattice of graphene, could be a crucial issue for the prediction of high-temperature creep resistance. Further work must be conducted to elucidate the local chemistry bonds involved in the ceramic composite at high temperatures.

## 4. Conclusions

The microstructure is constant during the creep at high temperatures and the deformation mechanism is grain boundary sliding.

The analysis of the high temperature mechanical properties allows for the conclusion that reduced graphene oxide does not provide an improvement of more than an order of magnitude in comparison to pure alumina, but it is potentially attractive in the range of nanocomposites or very high temperatures (from 1250 °C).

The reduced graphene oxide reinforced zirconia composites are, inside the experimental uncertainty, similar to pure zirconia regarding high-temperature creep properties. 

The ability of the carbon phase to “wet” the grain boundaries and distribute homogeneously (watching the rheological behavior, the fracture surfaces and the same positions of the peaks of the Raman spectra in different points of the samples) during deformation is a critical element to predict the high-temperature plasticity.

## Figures and Tables

**Table 1:** Grain sizes and shape factors for all the samples of alumina and its composites before and after deformation.

Sample	Grain Size (μm) As-Sintered	Grain Size (μm) After Creep	Shape Factor As-Sintered	Shape Factor After Creep
A	0.6 ± 0.3	1.7 ± 0.8	0.7 ± 0.1	0.7 ± 0.1
A2rGO	0.7 ± 0.3	1.0 ± 0.4	0.7 ± 0.1	0.7 ± 0.1
A6.7rGO	1.4 ± 0.6	2.4 ± 1.2	0.7 ± 0.1	0.7 ± 0.1

**Table 2:** Raman shifts (in cm^−1^) of the different peaks in the Raman spectra of alumina composites and ratio of the intensities of the D and G peaks.

Samples	α-Al_2_O_3_ Peak (cm^−1^)	α-Al_2_O_3_ Peak (cm^−1^)	D Peak (cm^−1^)	G Peak (cm^−1^)	2D Peak (cm^−1^)	D + G Peak (cm^−1^)	I_D_/I_G_
A2rGO powder	380	418	1350	1595	2601	2932	1.18 ± 0.03
A2rGO sintered specimen	378	420	1354	1602	2625	2886	0.86 ± 0.06
A2rGO after creep	375	414	1353	1592	2687	2904	0.71 ± 0.02
A6.7rGO powder	377	414	1350	1598	2599	2915	1.07 ± 0.01
A6.7rGO sintered specimen	380	420	1351	1586	2699	2920	0.41 ± 0.01
A6.7rGO after creep	380	423	1353	1587	2699	2926	0.70 ± 0.01

**Table 3:** Raman shifts (in cm^−1^) of the different peaks in the Raman spectra of zirconia composites and ratio of the intensities of the D and G peaks.

Samples	ZrO_2_ Peak (cm^−1^)	ZrO_2_ Peak (cm^−1^)	ZrO_2_ Peak (cm^−1^)	ZrO_2_ Peak (cm^−1^)	D Peak (cm^−1^)	G Peak (cm^−1^)	2D Peak (cm^−1^)	D + G Peak (cm^−1^)	I_D_/I_G_
Z2rGO powder	261	332	473	639	1354	1595	-	2927	1.25 ± 0.01
Z2rGO sintered specimen	261	328	466	642	1338	1590	2648	2900	1.14 ± 0.04
Z2rGO after creep	261	322	463	644	1350	1596	2691	2917	0.86 ± 0.02
Z6.7rGO powder	261	331	473	638	1350	1590	-	2926	1.19 ± 0.02
Z6.7rGO sintered specimen	261	325	466	642	1341	1593	2688	2921	1.02 ± 0.04
Z6.7rGO after creep	261	317	463	644	1351	1589	2699	2935	0.81 ± 0.02

## Data Availability

The data presented in this study are available on request from the corresponding authors.
